# Monolithic Papain-Immobilized Enzyme Reactors for Automated Structural Characterization of Monoclonal Antibodies

**DOI:** 10.3389/fmolb.2021.765683

**Published:** 2021-11-09

**Authors:** Francesca Rinaldi, Sara Tengattini, Gloria Brusotti, Giuseppe Tripodo, Benjamin Peters, Caterina Temporini, Gabriella Massolini, Enrica Calleri

**Affiliations:** ^1^ Department of Drug Sciences, University of Pavia, Pavia, Italy; ^2^ Instrumental Analytics R&D, Merck KGaA, Darmstadt, Germany

**Keywords:** monoclonal antibodies, analytical characterization, immobilized enzyme reactor (IMER), monolithic supports, polymerized high internal phase emulsions (polyHIPEs), papain, LC-MS, middle-up analysis

## Abstract

The characterization of monoclonal antibodies (mAbs) requires laborious and time-consuming sample preparation steps before the liquid chromatography–mass spectrometry (LC-MS) analysis. Middle-up approaches entailing the use of specific proteases (papain, IdeS, etc.) emerged as practical and informative methods for mAb characterization. This work reports the development of immobilized enzyme reactors (IMERs) based on papain able to support mAb analytical characterization. Two monolithic IMERs were prepared by the covalent immobilization of papain on different supports, both functionalized via epoxy groups: a Chromolith® WP 300 Epoxy silica column from Merck KGaA and a polymerized high internal phase emulsion (polyHIPE) material synthesized by our research group. The two bioreactors were included in an in-flow system and characterized in terms of immobilization yield, kinetics, activity, and stability using Nα-benzoyl-L-arginine ethyl ester (BAEE) as a standard substrate. Moreover, the two bioreactors were tested toward a standard mAb, namely, rituximab (RTX). An on-line platform for mAb sample preparation and analysis with minimal operator manipulation was developed with both IMERs, allowing to reduce enzyme consumption and to improve repeatability compared to in-batch reactions. The site-specificity of papain was maintained after its immobilization on silica and polyHIPE monolithic supports, and the two IMERs were successfully applied to RTX digestion for its structural characterization by LC-MS. The main pros and cons of the two supports for the present application were described.

## 1 Introduction

Biopharmaceutical market is rapidly growing, with monoclonal antibodies representing the most widespread products. Their therapeutic indications include a large variety of diseases, such as cancer, inflammation, diabetes, cardiovascular and genetic disorders, autoimmune diseases, and infections. Differently from small molecules, mAb drugs present heterogeneous and complex structures, and their production and characterization require the development of challenging and long processes (Sandra et al., 2014). The complexity of these macromolecules implies the investigation of several critical quality attributes (CQAs) with the consequent application of appropriate methods for their analytical control at intact, subunit, peptide, amino acid, and glycan levels (Fekete et al., 2013; Sandra et al., 2014).

Standard methods for mAb quality control usually include long sample preparation procedures with extensive sample manipulation. Therefore, faster and simpler methods are needed, especially for a rapid monitoring of the different steps involved in development and production processes.

Most of the current analytical methods for mAb in-depth structural characterization entail a preliminary structural simplification by enzymatic treatments due to the large size of these molecules (around 150 kDa) and the difficult acquisition of information from mAb intact analysis. Antibody digestion has been predominantly performed with trypsin (bottom-up approach), which is highly specific, easily available, and simple to use (Moore et al., 2016; Naldi et al., 2018). The small peptides obtained are then analyzed by liquid chromatography coupled to mass spectrometry (LC-MS) by peptide mapping. However, the time-consuming data analysis can limit the application in routine monitoring of product quality. Recently, the middle-up approach has been suggested as an alternative digestion method to overcome the limitations of conventional peptide mapping and to solve the challenges related to the analysis of intact antibodies. This approach includes the formation of 25–50 kDa mAb fragments by enzymatic treatment and/or reduction of disulfide bridges, followed by their separation and identification by analytical techniques such as LC-MS or capillary electrophoresis (CE)-MS. The structural simplification facilitates the interpretation of MS spectra, as well as the characterization of mAb isoforms, post-translational modifications, and glycosylation profiles compared to intact mAb analysis (Faid et al., 2018; Michalikova et al., 2019). The proteases most commonly used in the middle-up approach to generate mAb fragments are papain (Adamczyk et al., 2000) and, more recently, the immunoglobulin G–degrading enzyme from *Streptococcus pyogenes* or IdeS (Sjögren et al., 2016). Other enzymes such as pepsin, Lys-C, or IgdE have also been employed (Zhang et al., 2009; Faid et al., 2018).

Recently, a commercial IdeS column from Genovis™ was applied to the on-line digestion and characterization of mAbs (Camperi et al., 2020). However, IdeS protease is a particularly expensive enzyme in both its free and immobilized forms, while papain is more affordable and easily available. Therefore, papain was selected for the present work due to its greater suitability for an exploratory study. In addition, its different site-specificity compared to IdeS might provide further information for a comprehensive mAb characterization.

Papain is a non-specific thiol-endopeptidase that cleaves peptide bonds in the hinge region of mAbs. This treatment allows to obtain three fragments with a molecular weight of around 50 kDa, resulting in a more straightforward MS analysis compared to the intact mAb. The use of free papain presents some limitations, including the propensity to auto-digestion, the formation of adducts with mAbs, and the formation of heterogeneous fragments if the digestion conditions are not properly controlled. Papain immobilization on a solid carrier can prevent these problems and enable a better control of reaction conditions, an enhancement in enzyme stability, and an improved reaction repeatability (Thermo Fisher Scientific, 2021). Moreover, enzyme immobilization on chromatographic supports (flow-through immobilized enzyme reactors, IMERs) allows the coupling to LC-MS systems, thus increasing analysis throughput and automation.

The selection of a suitable support for enzyme immobilization is a crucial aspect to consider in order to preserve enzyme activity and promote enzyme–substrate interactions. Macromolecules such as antibodies require highly porous supports to interact with immobilized enzymes. In this regard, monolithic materials represent interesting supports for enzyme immobilization, due to their highly interconnected porous structure (Vlakh and Tennikova, 2013). Chromolith® Widepore columns are monolithic silica supports characterized by a peculiar bimodal pore structure presenting µm-sized macropores and nm-sized mesopores suitable for macromolecules (Merck KGaA, 2021). Another interesting monolithic support is represented by polymerized high internal phase emulsions (polyHIPEs), polymeric materials typically obtained from water in oil emulsions, in which the internal water phase represents the main constituent (higher than 70% v/v) and the external phase is composed of hydrophobic monomers. The polymerization of the external phase and the subsequent removal of the internal phase yield a monolithic material with a high degree of porosity and internal interconnections, presenting two types of macropores (throats and voids) with dimensions in the µm order. The possibility to use different functional monomers and polymerization techniques makes these polymers extremely versatile and suitable for several applications (Brusotti et al., 2016; Tripodo et al., 2018; Corti et al., 2019).

Other key features to evaluate for IMER preparation and characterization are its activity, kinetics, specificity, and stability. The maintenance of enzyme cleavage specificity after immobilization is particularly important when dealing with macromolecules, in order to avoid the production of unexpected digestion products and to guarantee sample quality. Enzyme specificity is generally assessed by MS analysis; the two most convenient MS ion sources for mAb characterization are matrix-assisted laser desorption/ionization (MALDI) and electrospray ionization (ESI). In order to study IMER activity and specificity, an on-line setup including the IMER, an LC separation system, and an ESI-MS detection can be established, while the use of a MALDI source requires an off-line MS analysis of the digested samples.

The aim of the work was the development of a papain-IMER to be included in an analytical platform to simplify and automate mAb analysis and characterization. Two monolithic supports for enzyme immobilization were compared: a commercial Chromolith® WP300 Epoxy silica column and an innovative polyHIPE material produced by our research group. An on-line system for automated mAb digestion and analysis was developed, and the two IMERs were characterized in terms of immobilization yield, activity, kinetics, specificity, and stability, highlighting pros and cons of the two supports. The digestion of the model antibody rituximab (RTX) was investigated to provide a proof of concept of the applicability of the platform in mAb analytical characterization.

## 2 Materials and Methods

### 2.1 Chemicals and Reagents

Butyl acrylate, glycidyl methacrylate, trimethylolpropane triacrylate (TMPTA), potassium persulfate, N,N,N′,N′-tetramethylethylenediamine (TEMED), methanol, glycine, Nα-benzoyl-L-arginine ethyl ester (BAEE), Nα-benzoyl-DL-arginine 4-nitroanilide hydrochloride (BAPNA), papain from papaya latex, Trizma® base (Tris), and ethylenediaminetetraacetic acid (EDTA) were purchased from Sigma-Aldrich (Milan, Italy). Synperonic PE/L121 was kindly provided by Croda Italiana SpA (Mortara, Italy). Tetrahydrofuran (THF), acetonitrile, and L-cysteine were from PanReac AppliChem ITW Reagents (Cinisello Balsamo, Italy). Potassium dihydrogen phosphate and formic acid were from Carlo Erba (Cornaredo, Italy).

Deionized water was obtained from a Milli-Q® Integral purification system from Merck KGaA (Darmstadt, Germany).

Rituximab (RTX) was purchased as MabThera from Roche (Basel, Switzerland).

Chromolith® Flash WP300 Epoxy silica columns of 4.6 × 25 mm were kindly provided by Merck KGaA (Darmstadt, Germany), while Omnifit® EZ-Solvent Plus glass columns of 10 × 100 mm by Diba Industries, Ltd., were purchased from Sepachrom-Mega Srl (Rho, Italy).

All reagents were of analytical grade.

### 2.2 Synthesis of the polyHIPE Support

The polyHIPE support was prepared following a previously described procedure (Brusotti et al., 2016; Tripodo et al., 2018; Corti et al., 2019). Briefly, the oil phase was obtained by mixing 1.02 ml of Synperonic PE/L121, 4.30 ml of butyl acrylate, 1.72 ml of glycidyl methacrylate, and 0.96 ml of TMPTA in a two-neck flask. The water phase, composed by 272 mg of potassium persulfate in 32 ml of nitrogen-degassed water, was added into the flask drop by drop using a dropping funnel. The addition of the water phase was carried out under nitrogen flow and continuous stirring at 300 rpm. After the addition, the system was maintained under nitrogen and stirring (400 rpm) for 1 h. A portion of the emulsion (around 10 ml) was transferred into a PE syringe connected by a female Luer coupler to a second syringe pre-filled with 70 μl of TEMED. The two components were mixed and promptly transferred into an Omnifit® EZ-Solvent Plus glass column of 10 × 100 mm to obtain a 10 × 9 mm chromatographic bed. The Omnifit® glass column is equipped with adjustable plungers to adapt to the polymer dimensions in order to avoid alternative flow paths. The *in situ* polymerization was carried out in the glass column for 24 h at room temperature. Then, the column was connected to a chromatographic system and washed by flowing methanol and subsequently THF at a maximum flow rate of 0.5 ml/min. The monolithic column was then stored in THF at room temperature until the immobilization procedure.

### 2.3 IMER Preparation

Papain was immobilized on a Chromolith® Flash WP300 Epoxy silica column (4.6 × 25 mm) and on a polyHIPE support polymerized as described in Section 2.2 in an Omnifit® EZ-Solvent Plus glass column (10 × 100 mm). Papain immobilization in the two columns was carried out by adapting *in situ* procedures developed by our group (Temporini et al., 2006; Tripodo et al., 2018). Due to the poor papain solubility in the described immobilization buffer, the protocol was adjusted by solubilizing the enzyme in a buffer with a lower molarity (50 mM phosphate buffer, pH 7.0).

The columns were included in a chromatographic system and washed with water (for 10 min at 0.5 ml/min for the silica support and for 30 min at 0.3 ml/min for the polyHIPE support). Then, an equilibration step was performed by flushing 50 mM phosphate buffer, pH 7.0, through the columns for at least 10 column volumes. A 0.5 mg/ml papain solution in 9.5 ml of immobilization buffer was recirculated into the columns (for 4 h at 0.5 ml/min for silica, for 24 h at 0.3 ml/min for polyHIPE), reversing the support at fixed times for 4 h (every 15 or 20 min for the silica and polyHIPE supports, respectively). Then, the silica column only was washed overnight with 10 mM phosphate buffer, pH 6.0, at 0.05 ml/min. The unreacted epoxide groups of the two supports were blocked by a 1 M glycine solution in 50 mM phosphate buffer, pH 7.0 (pumped into the silica column for 2 h at 0.5 ml/min and into the polyHIPE column for 40 min at 0.3 ml/min). The IMERs were washed with 50 mM phosphate buffer, pH 7.0, and stored at 4°C whenever not in use.

In addition, two blank columns were prepared as negative controls by performing only the endcapping step on new Chromolith® Flash WP300 Epoxy silica and polyHIPE columns with the same dimensions.

The immobilization yield was estimated spectrophotometrically at 280 nm for both IMERs. A papain calibration curve was built using the pre-immobilization solution of each IMER (five points, concentration range 16–500 μg/ml). The concentration of papain solutions after the immobilization procedure was calculated from the calibration curves, and the immobilization yield was estimated from the ratio between papain amount after and before the procedure.

### 2.4 Enzymatic Reaction Conditions

Digestion conditions were studied in solution for the three substrates RTX, BAEE, and BAPNA starting from literature protocols (Sigma-Aldrich, 2002; Lin et al., 2016).

For RTX, 100 µl of a 0.5 mg/ml mAb solution in different reaction buffers was incubated with papain in a 50/1 w/w ratio (1 µg of free papain). The investigated reaction buffers included 100 mM Tris-HCl, pH 7.6, 4 mM EDTA and 5 mM L-cysteine (Lin et al., 2016); 10 mM Tris, pH 6.2; and 10 mM Tris, 4 mM EDTA, and 5 mM L-cysteine, pH 6.2. Reactions were performed at 37°C (Lin et al., 2016). The reaction in 10 mM Tris, 4 mM EDTA, and 5 mM L-cysteine, pH 6.2, was carried out also at 25°C (Sigma-Aldrich, 2002).

For BAEE and BAPNA, 1 ml of a 0.1 mM substrate solution in the reaction buffer (10 mM Tris, 4 mM EDTA, and 5 mM L-cysteine, pH 6.2) was incubated at room temperature with 8 µg of free papain.

Reaction mixtures were incubated for 24 h and monitored by HPLC-UV at fixed times as described in Section 2.7.

### 2.5 Kinetic Studies

#### 2.5.1 In-Solution Digestion

Kinetic studies were performed in solution by the incubation of 10 µg of free papain with increasing amounts of BAEE (1, 2, 5, 10, 15, 20, 40 mM) in 1 ml of 10 mM Tris, 4 mM EDTA, and 5 mM L-cysteine, pH 6.2. Three replicate reactions were performed for each concentration, for 5 min at room temperature.

Papain’s specific activity was defined by incubating the same amount of free enzyme (10 µg) with 1 ml of a 100 mM BAEE solution in the reaction buffer. The reaction was carried out for 5 min at room temperature, and three replicates were performed.

After the incubation time, reactions were blocked by the addition of 30 mM iodoacetamide and samples were analyzed by HPLC-UV (Section 2.7) after appropriate dilution. Reaction rates were calculated as the ratio between Nα-benzoyl-L-arginine (BA) produced µmol (derived from the HPLC-UV analyses) and reaction time (5 min).

The Prism 9 software (GraphPad) was used to calculate the kinetic parameters V_max_ (maximal reaction rate, μmol/min) and K_m_ (Michaelis–Menten constant, mM) by a non-linear regression and the Michaelis–Menten enzyme kinetics equation; the applied model was Y = V_max_*X/(K_m_ + X). For the turnover number k_cat_, the model Y = Et*k_cat_*X/(K_m_ + X) was applied by choosing the k_cat_ equation in Prism. Et indicates the concentration of enzyme catalytic sites.

Specific activity was calculated considering the papain unit (U) definition: one unit is the amount of enzyme able to hydrolyze 1.0 μmol of BAEE per minute at pH 6.2, 25°C (Sigma-Aldrich, 2002).

#### 2.5.2 IMER In-Flow Digestion

Kinetic studies on monolithic silica- and polyHIPE-IMERs were carried out in-flow by injecting 20 µl BAEE solutions at increasing concentrations (15, 40, 60, 80, 100, 200, 300, 400, 500 mM) using a mobile phase composed by the reaction buffer (10 mM Tris, 4 mM EDTA, and 5 mM L-cysteine, pH 6.2) and a flow rate of 0.3 ml/min, resulting in a residence time of 1.384 min for the silica and 2.355 min for the polyHIPE bioreactor. Reactions were performed at room temperature, and eluates were collected at the IMER outlet for 30 min.

Each eluate was analyzed off-line by HPLC-UV as described in Section 2.7, after appropriate dilution when required. Reaction rates were calculated as the ratio between BA produced µmol (derived from the HPLC-UV analyses) and substrate residence times in each IMER.

Kinetic parameters were calculated by Prism software as specified for in-solution reactions (Section 2.5.1).

Specific activity was calculated as the ratio between the obtained V_max_ and the amount of immobilized enzyme on each IMER.

### 2.6 IMER Stop-Flow Digestion

#### 2.6.1 BAEE

The stop-flow digestion approach by the two IMERs was first tested on the BAEE substrate. Three replicate reactions were performed by the injection and overnight incubation in each IMER of 100 μl of a 20 mM BAEE solution in the reaction buffer (10 mM Tris, 4 mM EDTA, and 5 mM L-cysteine, pH 6.2) at room temperature. In parallel, 100 µl of a BAEE solution with the same composition was incubated in solution with 1 µg of free papain and in the blank columns to compare the digestion yields. Elution from the IMERs and blank columns was carried out by flowing the reaction buffer at 0.3 ml/min for 30 min. After incubation, in-solution reaction mixtures were diluted as the eluates, and all the samples were analyzed by HPLC-UV as described in Section 2.7.

#### 2.6.2 RTX

RTX was digested by applying the stop-flow approach setup on BAEE. Three replicate reactions were performed by the injection and overnight incubation in each IMER of 100 μl of a 0.5 mg/ml RTX solution in the reaction buffer (10 mM Tris, 4 mM EDTA, and 5 mM L-cysteine, pH 6.2) at room temperature. In parallel, 100 µl of a RTX solution with the same composition was incubated in solution with 1 µg of free papain (50/1 w/w mAb/enzyme ratio) and in the blank columns to compare the digestion yields. Elution from the silica-IMERs and blank silica column was carried out by flowing the reaction buffer at 0.3 ml/min for 30 min, while for the polyHIPE supports, a solution of 90/10 v/v reaction buffer/methanol was employed for the elution. After incubation and elution, samples were off-line analyzed by HPLC-UV (Section 2.7).

### 2.7 Instrumentation and Chromatographic Conditions

Chromatographic analyses of BAEE, BAPNA, and RTX samples were performed by an Agilent HPLC series 1200 system (Santa Clara, CA, United States), equipped with a mobile phase on-line degasser, quaternary pump, autosampler, column thermostated compartment, and diode array detector.

BAEE and BAPNA digestions were monitored using a Symmetry C18 column (4.6 × 75 mm, 3.5 μm, 100 Å) from Waters (Milford, CT, United States). The separation of each substrate from its product was performed by the use of mobile phases composed of water (A) and acetonitrile (B), both acidified with 0.1% v/v formic acid, a gradient elution, an injection volume of 10 μl, a flow rate of 1 ml/min, and a column temperature of 25°C. Gradient conditions and detection wavelengths were adapted to each substrate–product pair. The BAEE analytical method entailed the use of a gradient elution from 5 to 30% B in 6 min and a wavelength of 225 nm, while BAPNA and its digestion product were separated by a 20–45% B gradient in 6 min and were detected at 310 (BAPNA) and 410 nm (digestion product). Digestion yields were calculated as the ratio between the product area and the sum of substrate and product areas.

RTX samples were analyzed on an AdvanceBio RP-mAb C4 column (4.6 × 50 mm, 3.5 µm, 450 Å) from Agilent Technologies (Santa Clara, CA, United States), by using 0.1% v/v formic acid in water (A) and 0.1% v/v formic acid in acetonitrile (B) as mobile phases, a gradient elution, a flow rate of 1 ml/min, a column temperature of 70°C, and a wavelength of 280 nm. Gradient elution was carried out under the following conditions: 2–25% B in 2 min, followed by 25–60% B in 14 min, 60–95% B in 0.5 min, and an isocratic phase at 95% B for 5 min. An injection volume of 10 µl was used for the 0.5 mg/ml RTX solutions (intact or digested RTX), while the volume was increased to 1 ml for the IMER digestions (as a result of the sample dilution during the elution). Digestion yields were calculated as the ratio between the digested mAb area after incubation and the intact mAb area before incubation, while recoveries resulted from the ratio between the digested + intact mAb total area after and before incubation.

For MS detection, a X500 QTOF instrument from Sciex was used by applying the following parameters: curtain gas 40 psi, ion source gas 1 45 psi, ion source gas 2 55 psi, temperature 450°C, polarity positive, ion spray voltage 5500 V, CAD gas 7, time bins to sum 6, TOF start mass 600 Da, TOF stop mass 3,000 Da, accumulation time 1 s, declustering potential 150 V, and collision energy 10 V. Data processing and deconvolution of mAb fragment spectra were performed using Explorer for SCIEX OS software. Fragment attribution was carried out comparing the experimental value with the one calculated from the RTX amino acid sequence by mMass open-source software (version 5.5).

### 2.8 On-Line mAb Digestion and Analysis

**FIGURE 1:**
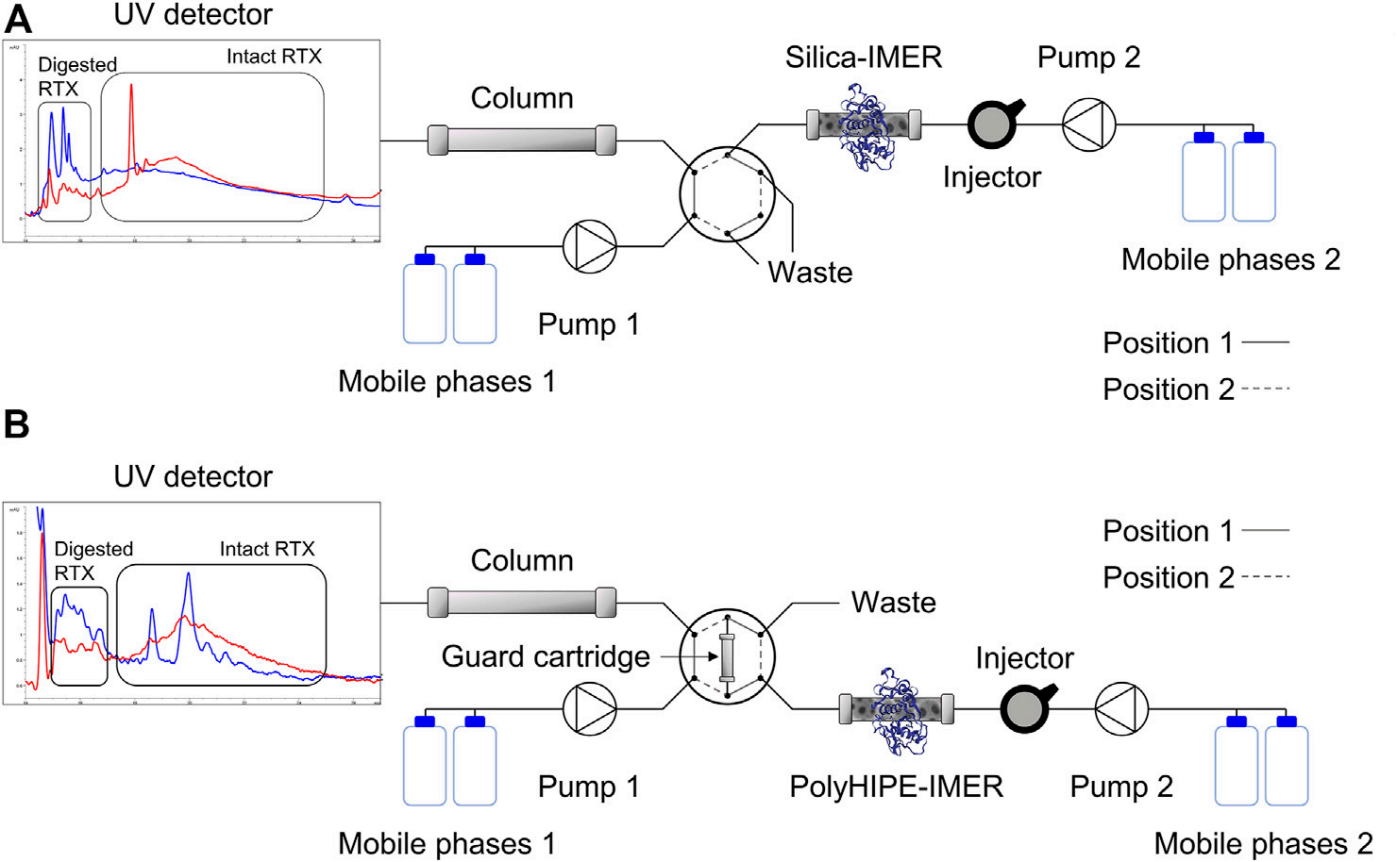
On-line system for automated mAb digestion and analysis with the silica-IMER **(A)** and polyHIPE-IMER **(B)**. Chromatograms of the RTX sample before (red) and after (blue) incubation in the IMERs are shown. A larger version of the chromatograms is included in the Supplementary Material.

For the on-line RTX digestion and analysis, two chromatographic systems were used (Figure 1). System 1 consisted of an Agilent HPLC series 1100 system equipped with a mobile phase on-line degasser, quaternary pump, column thermostated compartment, and variable wavelength detector, while system 2 was composed of a manual injector, a mobile phase on-line degasser, and a quaternary pump from Agilent. The two systems can be independent or connected in series through the switching of a six-port Rheodyne valve automatically controlled. The AdvanceBio RP-mAb C4 column (4.6 × 50 mm, 3.5 µm, 450 Å) from Agilent was included in the first system and the IMERs in the second. For the on-line analysis of polyHIPE-IMER digests, a guard cartridge was attached to the automatic Rheodyne valve and connected alternatively to the IMER for the eluate collection or to the column for the analysis. A Fortis BIO-C4 (4.0 × 10 mm, 300 Å) guard cartridge kindly provided by CPS Analitica (Milano, Italy) was used.

For the system including the silica-IMER (Figure 1A), on-line digestion and analysis of RTX samples were performed according to the following procedure:

Step 1 (valve in position 1): loading of a 0.5 mg/ml RTX solution (20 µl) in the IMER. The mobile phase consisted of the reaction buffer (10 mM Tris, 4 mM EDTA, and 5 mM L-cysteine, pH 6.2); the flow rate was set at 0.3 ml/min and stopped after 0.4 min from the injection for the stop-flow overnight incubation (around 16 h).
Step 2 (valve in position 2): transfer of the IMER eluate to the analytical column. After the stop-flow incubation, the reaction buffer was flowed through the IMER at 0.3 ml/min, and the reaction mixture was transferred directly to the column, which was connected in line with the IMER for 6 min.
Step 3 (valve in position 1): analysis of the IMER eluate. The analytical column was connected again to the system including its mobile phases (A: 0.1% v/v formic acid in water; B: 0.1% v/v formic acid in acetonitrile) and was washed for 3 min with 98% A to remove the excess of salts; then, the gradient developed for the off-line RTX analyses was applied to separate and quantify the substrate and products.

For the on-line system including the polyHIPE-IMER (Figure 1B), a slightly different procedure was applied:

Step 1 (valve in position 2): loading of a 0.5 mg/ml RTX solution (20 µl) in the IMER. The mobile phase consisted of the reaction buffer (10 mM Tris, 4 mM EDTA, and 5 mM L-cysteine, pH 6.2); the flow rate was set at 0.3 ml/min and stopped after 0.5 min from the injection for the stop-flow overnight incubation (around 16 h).
Step 2 (valve in position 1): transfer of the IMER eluate to the guard cartridge. After the stop-flow incubation, a solution of 90/10 v/v reaction buffer/methanol was flowed through the IMER at 0.3 ml/min, and the reaction mixture was transferred to the guard cartridge, which was connected in line with the IMER for 6 min.
Step 3 (valve in position 2): analysis of the IMER eluate. The guard cartridge was connected to the system including the analytical column and its mobile phases (A: 0.1% v/v formic acid in water; B: 0.1% v/v formic acid in acetonitrile) and was washed for 3 min with 98% A to remove the excess of salts; then, the gradient developed for the off-line RTX analyses was applied to separate and quantify the substrate and products.

A complete cycle of the on-line automated digestion and analysis workflow requires around 16.5 h.

## 3 Results

### 3.1 In-Solution Assays: Setup of the Reaction Conditions and Kinetic Studies

Papain activity was first investigated in solution to define the best digestion conditions for the intended purpose. Since the aim of the work is the application of papain-IMERs to antibody digestions, reaction conditions were studied on a commercial mAb, namely, RTX.

Starting from a digestion in 100 mM Tris-HCl, pH 7.6, 4 mM EDTA and 5 mM cysteine buffer at 37°C as described in a Thermo Fisher Scientific Application Note (Lin et al., 2016), the influence of reaction buffer composition on the digestion yield was evaluated. As expected, the presence of EDTA and cysteine in the buffer was found to be essential for papain activity since their removal from the reaction buffer resulted in a complete suppression of the digestion. Instead, the reduction of Tris concentration to 10 mM and the consequent pH change to 6.2 maintaining the same concentrations of EDTA and cysteine were not found to have a significant impact on the digestion yield. A reduction in the yields over time was observed at 37°C in both 100 mM Tris-HCl, pH 7.6, 4 mM EDTA and 5 mM cysteine buffer and 10 mM Tris, 4 mM EDTA, and 5 mM cysteine buffer, pH 6.2, and it was attributed to the degradation of digestion products; therefore, a lower temperature (25°C) was tested for the digestion, and a higher yield was obtained after 24 h, probably due to the greater stability of the products in these conditions (Table 1). Thus, the selected reaction conditions for further experiments were a reaction buffer composed of 10 mM Tris, 4 mM EDTA, and 5 mM L-cysteine, pH 6.2, and room temperature.

**TABLE 1:** Results of the 24 h incubation of 100 µl of 0.5 mg/ml RTX with 1 µg of free papain in different conditions. Digestion yields are calculated as the ratio between the digested mAb area after incubation and the intact mAb area before incubation obtained from the HPLC-UV analysis of the reaction mixtures.

Reaction conditions	Digestion yield (%)
100 mM Tris-HCl, pH 7.6, 4 mM EDTA and 5 mM cysteine buffer; 37°C	19.70
10 mM Tris, pH 6.2; 37°C	—
10 mM Tris, 4 mM EDTA, and 5 mM cysteine buffer, pH 6.2; 37°C	21.98
10 mM Tris, 4 mM EDTA, and 5 mM cysteine buffer, pH 6.2; 25°C	51.29

The selected conditions were tested also on the in-solution digestion of the standard papain substrates BAEE and BAPNA, giving digestion yields of 74.92 and 12.88%, respectively (24 h incubation of a solution of 0.1 mM substrate in the reaction buffer with 8 µg of enzyme, final volume 1 ml).

**FIGURE 2:**
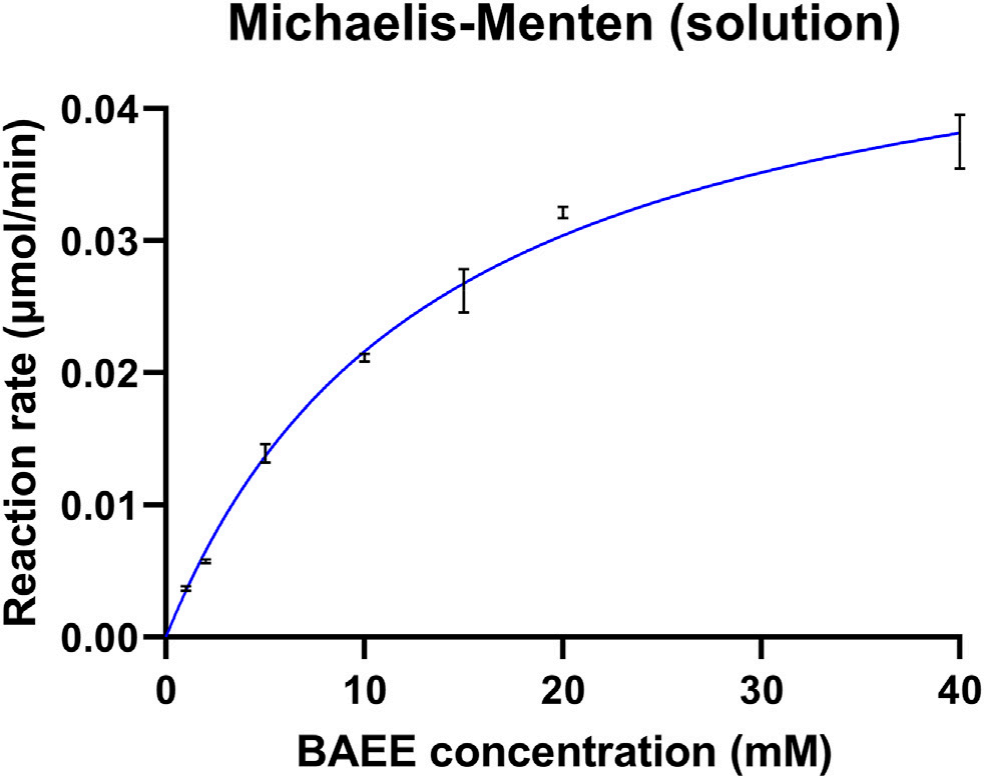
Kinetic profile of free papain. The enzyme was incubated with increasing concentrations of BAEE. Each point is the mean of three replicates, with error bars indicating ±standard deviation.

Enzyme kinetics was assessed in solution prior to papain immobilization. Kinetic studies were performed on the BAEE reference substrate since its digestion was significantly faster compared to BAPNA. Papain was incubated with increasing amounts of BAEE (1–40 mM, three replicates for each concentration) to define kinetic parameters, which are reported in Table 2. V_max_ expresses the maximum reaction rate achievable from the enzyme, K_m_ is the Michaelis–Menten constant and represents the substrate concentration allowing to reach half of the V_max_, and the turnover number k_cat_ corresponds to the times in which substrate molecules are converted into the product by each enzyme catalytic site per unit of time. Papain’s specific activity was calculated in the presence of a saturating substrate concentration (100 mM) and was found to be consistent with the supplier specifications (≥10 units/mg protein). One papain unit (U) is defined as the amount of enzyme able to hydrolyze 1.0 μmol of BAEE per minute at pH 6.2, 25°C (Sigma-Aldrich, 2002). Michaelis–Menten kinetics is shown in Figure 2. Kinetic parameters were calculated by the Prism 9 software (GraphPad) as reported in Section 2.5.1.

**TABLE 2:** Free papain kinetic parameters calculated using BAEE as the substrate.

V_max_ (µmol/min)	K_m_ (mM)	k_cat_ (min^−1^)	Specific activity (U/mg)
0.051	13.71	119.9	13.5

### 3.2 Preparation and Characterization of Papain-IMERs

After enzyme activity investigation by in-solution assays, two monolithic IMERs were prepared by the covalent immobilization of papain on two different supports, both functionalized *via* epoxy groups: a Chromolith® WP300 Epoxy silica column from Merck KGaA and a polyHIPE material synthesized by our research group as in Section 2.2. The two bioreactors were included in an in-flow system and characterized in terms of immobilization yield, kinetics, and activity, in order to compare the two monolithic supports.

#### 3.2.1 Immobilization Yield

Papain was immobilized on the silica and polyHIPE supports by adapting *in situ* procedures developed by our group (Temporini et al., 2006; Tripodo et al., 2018), as detailed in Section 2.3.

Immobilization yields were evaluated by a spectrophotometric assay carried out at 280 nm. For each IMER, a five-point calibration curve was obtained by serial dilutions of the papain pre-immobilization solution in the concentration range 16–500 μg/ml. The curves showed a good linearity (*y* = 1.7218*x* − 0.0303, *R* ^2^ = 0.9984 for papain solution used for the immobilization on the silica support and *y* = 1.1515*x* − 0.0009, *R* ^2^ = 0.9943 for papain used on the polyHIPE) and allowed to derive the concentration of the post-immobilization solutions. The percentage of immobilized enzyme was estimated by the ratio between the papain amount after and before the immobilization procedure, resulting in 15.19% on the silica support and 35.87% on the polyHIPE material. The immobilized papain amount and density per unit column volume are reported in Table 3.

**TABLE 3:** Main features of the silica- and polyHIPE-IMERs.

IMER	Internal volume (mm^3^)^a^	Immobilized enzyme (mg)	Enzyme density (µg/mm^3^)	V_max_ (µmol/min)	K_m_ (M)	k_cat_ (min^−1^)	Specific activity (U/mg)	Active enzyme density (U/mm^3^)
Silica	415.3	0.7	1.7	0.086	0.658	2.9	0.123	2.07 * 10^–4^
PolyHIPE	706.5	1.7	2.4	0.148	1.530	2.0	0.087	2.09 * 10^–4^

a Dimension of the support.

#### 3.2.2 Kinetic Studies

**FIGURE 3:**
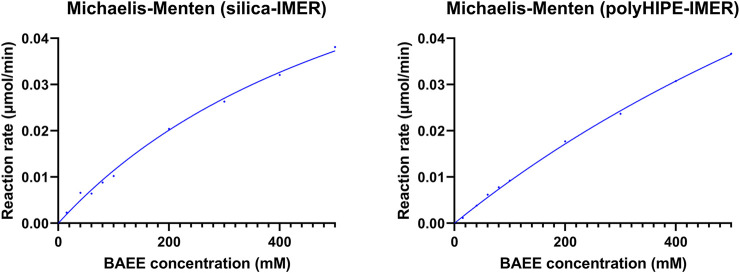
Kinetic profiles of papain immobilized on the silica and polyHIPE supports.

Michaelis–Menten studies were performed using the standard substrate BAEE to estimate kinetic parameters, which were calculated by the Prism 9 software (GraphPad) as reported in Section 2.5.2. In-flow reactions were carried out by a single passage through each IMER of BAEE solutions at different concentrations (15–500 mM) using a mobile phase composed by the reaction buffer (10 mM Tris, 4 mM EDTA, and 5 mM L-cysteine, pH 6.2) and a flow rate of 0.3 ml/min. Each eluate was collected at the IMER outlet for 30 min and analyzed off-line by HPLC-UV under the conditions described in Section 2.7 to define the BA production rate. Kinetic parameters and activity data derived for the silica- and polyHIPE-IMERs are reported in Table 3. Kinetic profiles are shown in Figure 3.

### 3.3 Application of Papain-IMERs to mAb Digestion

The characterized silica- and polyHIPE-IMERs were then applied to the digestion of the commercial mAb RTX. The antibody was injected in the flow-through IMERs using the same conditions set up for the kinetic studies. No digestion products were detected. This result was ascribed to the short substrate residence time in the IMERs (1.384 min for the silica and 2.355 min for the polyHIPE at a flow rate of 0.3 ml/min). This hypothesis is coherent with the slow kinetics observed for RTX digestion in solution (digestion yield ≈ 51% after the 24 h incubation of a 0.5 mg/ml RTX solution with 1 µg papain, Table 1), suggesting that a long substrate–enzyme contact time is needed for the digestion.

Therefore, a recirculation mode was tested by continuously pumping the reaction mixture through each bioreactor at a constant flow rate (0.3 ml/min). Reaction monitoring at fixed times revealed a reduction of intact mAb over time, which was not counterbalanced by a corresponding increase in digestion products indicating a possible non-specific interaction of RTX with the monolithic supports.

In order to explore all the options, a stop-flow approach was also tested to increase substrate-immobilized papain contact time.

The stop-flow approach was initially tested on the standard substrate BAEE to set up the incubation and elution conditions. The best results were obtained after the overnight incubation of 100 µl of a 20 mM BAEE solution, followed by the eluate collection by flowing the reaction buffer through the bioreactors for 30 min at 0.3 ml/min. Reactions performed in the two IMERs were compared with digestions of the same 20 mM BAEE solutions by 1 µg of free papain. In addition, blank columns were prepared by performing only the endcapping step of the immobilization procedure on a new silica or polyHIPE column, as described in Section 2.3. BAEE was incubated also in the blank columns as a negative control. Digestion yields were calculated as the ratio between the BA area and the BA + BAEE total area obtained from the off-line HPLC-UV analysis of the eluates (Table 4).

**TABLE 4:** Results of the overnight digestion of 100 µL of 20 mM BAEE by papain in solution and in IMERs.

Enzyme	Reaction mode	Analysis mode	Digestion yield % (mean ± SD)
Free	Solution	Off-line	28.46 ± 1.16
Silica-IMER	Stop-flow	Off-line	96.47 ± 3.09
None, blank silica column	Stop-flow	Off-line	1.42 ± 0.69
PolyHIPE-IMER	Stop-flow	Off-line	66.25 ± 1.79
None, blank polyHIPE column	Stop-flow	Off-line	1.48 ± 0.18

The stop-flow digestion conditions set up for BAEE were applied to RTX samples. A 0.5 mg/ml RTX solution (100 µl) was incubated overnight in the IMERs, in solution with free papain (positive control), and in the blank columns (negative control).

RTX incubation in silica-IMERs (1 and 2, prepared in the same way and containing the same amount of immobilized enzyme) resulted in satisfactory digestion yields, but also in an incomplete sample elution. In fact, the off-line HPLC-UV analysis of the eluates revealed that the substrate and products were not present at the expected concentration (Table 5). This result was attributed to a non-specific binding of RTX to the support, as suggested also by the low mAb recoveries in the eluates from the blank silica column (Table 5). Different strategies were tested for mAb desorption; elution was performed in the presence of a low percentage of organic solvent or changing the pH of the elution buffer to reduce substrate–support hydrophobic or ionic interactions, but none of the tested approaches allowed to collect the expected amount of substrate and products, and a decrease in digestion activity was observed after RTX incubation in the two silica-IMERs (see Section 3.5). Thus, it was not possible to perform replicate reactions.

**TABLE 5:** Results of the overnight digestion of 20 (on-line) or 100 µl (off-line) of 0.5 mg/ml RTX by papain in solution or in silica- and polyHIPE-IMERs. Results for the silica-IMERs derive from the two different bioreactors (1 and 2) obtained using this support.

Enzyme	Reaction mode	Analysis mode	Digestion yield % (mean ± SD)	Recovery % (mean ± SD)
Free	Solution	Off-line	29.59 ± 1.00	89.64 ± 2.22
Silica-IMER 1	Stop-flow	Off-line	5.25^a^	25.50^a^
Silica-IMER 2	Stop-flow	Off-line	21.34^a^	55.60^a^
Silica-IMER 2	Stop-flow	On-line	9.35^a^	36.45^a^
None, blank silica column	Stop-flow	Off-line	—	46.25 ± 1.71
PolyHIPE-IMER	Stop-flow	Off-line	2.10 ± 1.93	47.06 ± 4.31
PolyHIPE-IMER	Stop-flow	On-line	12.84 ± 3.23	79.54 ± 0.81
None, blank polyHIPE column	Stop-flow	Off-line	—	82.27 ± 1.94

a Value derived from a single measurement.

The adsorption of RTX on the hydrophobic polyHIPE material was more evident compared to the silica columns when using the reaction buffer for elution. Thus, the addition of a small percentage of an organic solvent to the elution buffer was investigated. Methanol was selected because it is one of the solvents employed in the polyHIPE washing procedure, and the typical swelling of the polymer associated with the use of this solvent is well-studied. The use of 10% methanol resulted beneficial for mAb elution, increasing the recovery from nearly 0% in the buffer alone to almost 50%. Therefore, for the polyHIPE-IMER, the elution was performed using a solution of 90/10 v/v reaction buffer/methanol that allowed to obtain repeatable yields and recoveries avoiding activity losses (Table 5; Section 3.5).

For both supports, reaction mixtures were collected after the overnight incubation by flowing the reaction buffer (with or without methanol) through the bioreactors for 30 min at 0.3 ml/min as for the BAEE substrate. Digestion yields and recoveries were derived from the off-line HPLC-UV analysis of the eluates as described in Section 2.7 and are reported in Table 5.

#### 3.3.1 On-Line mAb Digestion and Analysis

One of the final aims of this work was the development of an automated platform for the on-line digestion and analysis of mAbs. The working conditions of the on-line system were investigated on BAEE and then applied to RTX samples.

The BAEE standard substrate was used to define the sample injection volume and the time required to reach the IMER for sample incubation. Using the second silica-IMER, the best results were obtained with an injection volume of 20 µl and a stop-flow after 0.4 min from the injection for substrate incubation into the IMER. In these conditions, a 30 min incubation of BAEE 20 mM in the silica-IMER resulted in a digestion yield of 46.36%. For the polyHIPE-IMER, an injection volume of 20 µl and a stop-flow after 0.5 min from the injection allowed to reach the highest digestion yield (6.83% for the 30 min incubation of BAEE 20 mM in the IMER).

In addition, the conditions for sample transfer to the analytical column were defined based on the features of the two IMERs. Chromolith® columns can work at high pressures (up to 200 bar) (Merck KGaA, 2021), allowing for a direct transfer of the reaction mixture from the silica-IMER to the analytical column. On the contrary, since Omnifit® glass columns tolerate backpressures up to 40 bar (Diba Industries Ltd., 2016), it was necessary to include a guard cartridge in the on-line system for the collection of polyHIPE-IMER eluate.

The on-line platform was then applied to RTX samples (Figure 1). The digestion was carried out by injecting 20 µl of a 0.5 mg/ml RTX solution in the IMERs at 0.3 ml/min and stopping the flow rate for the overnight RTX incubation after 0.4 (silica-IMER) or 0.5 (polyHIPE-IMER) minutes from the injection, as defined on the small substrate. After the incubation time, the IMER was connected to the guard cartridge (polyHIPE-IMER) or directly to the analytical column (silica-IMER) through the switching of the six-port valve, and the reaction mixture was transferred by flowing the reaction buffer (with or without methanol) in the bioreactor for 6 min at 0.3 ml/min. The analysis started by the subsequent switch of the valve position allowing the flow of the mobile phases in the guard cartridge and/or analytical column. Experimental details are given in Section 2.8. The overnight incubation of RTX in the second silica-IMER resulted in a digestion yield of 9.35% and a recovery of 36.45%, while for the polyHIPE-IMER, a digestion yield of 12.84 ± 3.23% and a recovery of 79.54 ± 0.81% (mean of three replicate reactions) were obtained (Table 5).

### 3.4 Assessment of IMERs’ Specificity

**FIGURE 4:**
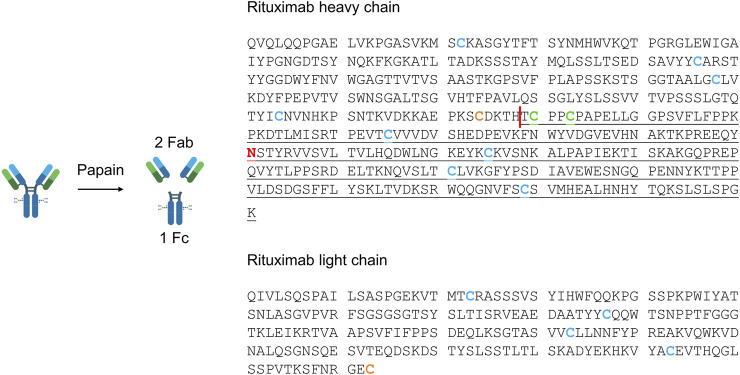
Amino acid sequence of rituximab heavy and light chains. The papain cleavage site is shown as a red vertical line. The Fab fragment is composed of the non-underlined amino acids of the heavy and light chains, while Fc residues are underlined. The glycosylation site is represented by the red asparagine in the Fc region. Cysteines included in intrachain (blue) and interchain (orange between light and heavy chains, green between two heavy chains) disulfide bonds are highlighted. The reaction scheme was created with BioRender.com.

The site-specificity of immobilized enzymes is a crucial feature to define in IMERs. Thus, papain’s specificity in the two IMERs compared to the soluble enzyme was assessed by LC-MS analyses. Papain is able to cleave peptide bonds in the hinge region generating three 50 kDa fragments: two Fab and one Fc (Figure 4). In RTX, the cleavage should occur between H_228_ and T_229_ residues of the heavy chain. Table 6 indicates fragment assignment in LC-MS analyses of reaction mixtures derived from RTX digestion by free and immobilized papain. The deconvoluted spectra obtained for each fragment are reported in the Supplementary Material.

**TABLE 6:** Assigned mAb fragments in LC-MS analyses of RTX digested by papain in solution and in the silica- and polyHIPE-IMERs. -K = cleaved C-terminal lysine; p = N-terminal pyroglutamate modification.

Fragment assignment		Theoretical average mass (Da)	Detected average mass (Da)		
			Solution	Silica-IMER	PolyHIPE-IMER
Fc/2-K	+G0F	26,377.3	26,379.0	26,378.5	26,378.6
	+G1F	26,539.5	26,541.2	26,540.6	26,540.5
	+G2F	26,701.6	26,703.1	26,702.8	—
pLC		23,034.4	23,034.9	23,035.4	23,034.8
Fc-K	+2G0F	52,754.7	52,755.9	52,757.4	52,754.8
	+1G0F+1G1F	52,916.8	52,917.8	52,918.9	52,917.4
	+1G0F+1G2F (or 2G1F)	53,079.0	53,080.1	53,082.4	53,079.8
	+1G1F+1G2F	53,241.1	53,242.4	53,244.7	—
	+2G2F	53,403.2	53,406.1	53,408.2	—
pFab		47,178.2	47,178.4	47,181.6	47,177.4
pFab–pLC		24,143.8	24,144.4	24,144.7	24,143.9

Besides the expected Fab and Fc, fragments derived from the breakage of disulfide bonds (Fc/2; light chain, LC; the portion that constitutes Fab together with LC, Fab-LC) were detected. Fab and LC were only found as carrying the N-terminal pyroglutamate modification (pFab and pLC). The Fc fragment and its half Fc/2 were detected as lacking the C-terminal lysine (Fc-K and Fc/2-K), and their glycosylation profile was consistent with literature data (Wang et al., 2013). The most abundant species correspond to glycoforms G0F, G1F, and G2F, containing a common core composed of four N-acetyl glucosamine, one fucose, and three mannose residues linked to 0, 1, or 2 galactose residues.

LC-MS analyses proved that immobilized papain maintained its site-specificity in polyHIPE- and silica-IMERs, since digestion products were detected at the expected molecular weights.

### 3.5 IMERs’ Stability

The possibility to use immobilized enzymes for multiple cycles and to achieve reproducible results represents key features for IMERs. Thus, IMER stability was monitored over time by the activity assay developed on the standard substrate BAEE using the stop-flow approach.

The activity of both silica-IMERs decreased significantly after RTX adsorption. The BAEE digestion yield changed from around 96% before RTX incubation (Table 4) to 3.99 and 7.39% after its incubation in the first and second silica-IMERs, respectively.

In contrast, to date, the polyHIPE-IMER retained its catalytic activity over 90 reactions in 12 months, giving a digestion yield of 66.20 ± 2.01% (mean of three replicate reactions) after 1 year from the immobilization; this result is comparable to the ones obtained on the fresh IMER (Table 4). A relative standard deviation of 2.57% was obtained across the six replicate reactions (three on the fresh IMER and three after 1 year from the immobilization).

## 4 Discussion

Monoclonal antibody therapy is having a significant impact on many diseases. The fast growth of mAb applications emphasizes the need to simplify and automate their laborious and time-consuming analytical characterization during development and production processes. Prompted by the requirement of sample enzymatic treatments for the investigation of the main mAb CQAs, the aim of the work was the development of a papain-IMER to be included in a model platform to support routine mAb analysis and characterization.

The development of a system for on-line digestion with IMERs requires a preliminary investigation of the reaction conditions. Starting from literature protocols, the concentration of the reaction buffer was decreased to minimize the risk of mAb precipitation in the in-flow system. The influence of temperature on the digestion yields was also considered to define the need of IMER thermostatation. The decrease in Tris concentration did not significantly impact on the yields, while a reduction in the reaction temperature resulted beneficial for substrate and product stability. Thus, reactions were performed in 10 mM Tris, 4 mM EDTA, and 5 mM L-cysteine buffer, pH 6.2, at room temperature. In addition, kinetic parameters of free papain were defined before its immobilization to assess the effect of the linkage to the supports on its activity.

Two monolithic supports were selected for papain immobilization, since these kinds of materials present a highly porous structure and a wide surface area particularly appropriate for macromolecules’ loading and interaction. A Chromolith® Widepore column from Merck KGaA, presenting wide mesopores (300 Å) suitable for antibody samples, was chosen as a reference material and was compared with a polymeric support (polyHIPE) synthesized by our research group, characterized by a highly interconnected structure with µm-sized macropores. The composition of the polyHIPE was optimized in preliminary studies, and the complete physico-chemical characterization of the material is described in previously published papers (Brusotti et al., 2016; Tripodo et al., 2018; Corti et al., 2019). Both the supports were functionalized with epoxy groups, so that papain was covalently bound via a stable multipoint immobilization.

The derived IMERs were included in an in-flow system for their characterization in terms of immobilization yield, kinetics, and activity. Two important parameters for IMER activity are the amount of immobilized enzyme, for which high values should translate into a greater enzyme/substrate ratio and reaction rate, and the enzyme density, which influences the accessibility of the substrate to the active sites (Temporini et al., 2006). A higher immobilization yield and enzyme density were obtained on the polyHIPE support. However, the higher K_m_, lower specific activity, and reduced k_cat_ of papain immobilized on this material revealed that a lower percentage of enzyme was immobilized in its active form compared to the silica support. This result can be ascribed to the high hydrophobicity of polyHIPE material, which promotes enzyme adsorption yielding a larger amount of immobilized macromolecule, but also leads to enzyme unfolding with a consequent reduction of activity in most cases (Mao et al., 2020). Although a direct comparison between kinetic parameters of free and immobilized enzymes is not possible due to the marked difference in terms of enzyme/substrate ratio and enzyme mobility, it was possible to observe a reduction in affinity to the substrate and activity per unit of enzyme after immobilization. In particular, a K_m_ increase of 50 (silica) or 100 (polyHIPE) folds, specific activity reduction of 100 (silica) or 150 (polyHIPE) folds, and k_cat_ decrease of 40 (silica) or 60 (polyHIPE) folds were obtained from the IMERs compared to the free enzyme. This might be due to changes in the enzyme structure and/or inaccessibility of active sites as a result of the covalent immobilization, but also to long immobilization times which can have a negative impact on enzyme activity (Mao et al., 2020). Nevertheless, the possibility to use IMERs for many digestion cycles allows to achieve greater yields and more reproducible reactions compared to free enzymes.

After their characterization, IMERs were applied to the digestion of the model antibody RTX. Conversely to that observed with the small substrate BAEE, in-flow reactions were not effective for the digestion of RTX. The slow reaction rate on this substrate requires a long enzyme–antibody contact time, so a stop-flow approach was selected for the digestion. The optimized experimental conditions resulted in BAEE digestion yields two or three times greater (for polyHIPE and silica, respectively) using IMERs compared to free papain. The improvement in the reaction rates due to the much higher enzyme/substrate ratio is one of the advantages of enzymes immobilized on the surface of a suitable carrier material, together with storage stability and ease of use. RTX incubation in the silica-IMERs resulted in an incomplete elution due to antibody–support interactions, which was not possible to reduce with the tested approaches. The adsorption of RTX led to a fast loss of IMERs' activity. Antibody adsorption was observed also into the polyHIPE-IMER, but the addition of a low percentage of methanol to the elution buffer allowed to increase mAb recovery maintaining IMER activity over time. Even if papain immobilized on the silica columns showed a greater activity on both the tested substrates, it turned out that polyHIPE is a better support for mAb reactions due to the easy reversibility of non-specific hydrophobic interactions and to the IMER stability over time (constant BAEE digestion yields were observed over 90 reactions in 12 months).

The possibility to include IMERs into on-line automated systems led us to develop a platform for mAb sample preparation and analysis with minimal operator manipulation. The operating conditions were set up on BAEE, and the on-line system was then applied to RTX structural simplification and analysis. The specifications of columns including silica and polyHIPE supports required a different system configuration for the two IMERs. In particular, the high backpressures tolerated by Chromolith® columns enabled a direct connection between the silica-IMER and the analytical column, while the higher fragility of the Omnifit® glass columns required the introduction of a guard cartridge in the system to mediate sample transfer from the polyHIPE-IMER to the analytical column. Differently from the off-line analysis of IMER-digested samples, in the on-line platform, all the sample incubated in the IMERs was transferred to the analytical system allowing for a reduction of the injection volume from 100 to 20 µl. The lower amount of RTX resulted beneficial for the digestion yield and recovery from the polyHIPE-IMER (around 13% yield and 80% recovery in the on-line system compared to 2 and 47% off-line). Instead, the results obtained from the silica-IMER confirmed the partial activity loss of this bioreactor.

The advantages of IMERs in terms of enzyme reusability and system automation encouraged us to apply them to RTX characterization by LC-MS, as a proof of concept of the applicability of the developed platform to mAb routine analysis and characterization. LC-MS analyses allowed to assign the identity of digestion products and to define the site-specificity of the IMERs compared to the free enzyme. The maintenance of enzyme cleavage specificity after immobilization is another critical feature to assess in IMERs, especially when applied to reactions involving macromolecules; both polyHIPE and silica supports proved to maintain papain site-specificity after immobilization, confirming their applicability in mAb analytical characterization. The loss of specificity can indeed generate unexpected digestion products not suitable for quality control analyses. The LC-MS characterization revealed that the expected digestion products were generated, together with a few fragments derived from the breakage of disulfide bonds. All the fragments identified in the sample digested by free papain were detected also in the reaction mixtures derived from the two IMERs, with minor differences due to the lower concentration of the IMER eluates. Fab and LC were only detected as carrying the N-terminal pyroglutamate modification, in accordance with the literature (Wang et al., 2013). This post-translational modification derives from the conversion of an N-terminal glutamine into a pyrrolidinone ring with the loss of ammonia and is reported as not significantly affecting mAb pharmacological properties, since the N-terminus is not a functionally relevant region (Liu et al., 2014). Instead, the Fc fragment and its half Fc/2 were detected as lacking the C-terminal lysine (Fc-K and Fc/2-K), another non-critical modification for mAb safety and efficacy. Differently from N-terminal pyroglutamate and C-terminal lysine modifications, glycosylation represents one of the most critical attributes in mAbs. The LC-MS analysis of digested samples by both papain-IMERs enabled the characterization of RTX glycosylation profile, presenting G0F, G1F, and G2F as the most abundant glycoforms, as described in the literature (Wang et al., 2013).

Summing up, the silica support was more appropriate for papain immobilization and maintenance of its active conformation but critical when using large substrates as mAbs; on the contrary, the polyHIPE material demonstrated a greater stability over time and emphasized IMER benefits when compared to in-solution reactions. In addition, the versatility of this polymeric material in terms of composition and scalability enables to adapt it to the desired application. The modulation of pore dimensions and polymer hydrophobicity should provide a better support for mAb samples. The main pros and cons of the two supports in mAb characterization are summarized in Table 7.

**TABLE 7:** Main advantages (pros) and disadvantages (cons) of the investigated supports applied to papain immobilization for mAb digestion and characterization.

Support	Pros	Cons
Silica	- Maintenance of papain activity after immobilization and higher digestion yields on the tested substrates	- mAb adsorption leading to a loss of activity and stability
	- Maintenance of papain site-specificity	
	- High tolerated backpressures and flow rates	- Non-customizable material
	- Possibility to include the IMER in an automated on-line system	
PolyHIPE	- Maintenance of papain site-specificity	- Partial papain activity loss after immobilization
	- Repeatability of the reactions and stability over time	- Backpressure limits
	- Possibility to include the IMER in an automated on-line system	
	- Versatility in terms of composition and scalability leading to a material tailored to the samples and applications of interest	
	- Applicability to mAb middle-up characterization

The study on the model antibody RTX provided a proof of concept for the applicability of the developed platform to the automated sample preparation and analysis of mAbs, valuable in various stages of their development and production. The ability of papain to cleave in a conserved region of immunoglobulins G (IgGs) and the maintenance of enzyme site-specificity after immobilization suggest that the platform should give consistent results for different IgGs. It must be pointed out that RTX is a particularly hydrophobic mAb (hydrophobicity = −0.414 as reported by the DrugBank Online Database). It can be expected that the analysis of more hydrophilic antibodies and the optimization of polyHIPE composition (use of more hydrophilic monomers) might reduce non-specific interactions and improve system performances. In addition, different enzymes (proteases, carboxypeptidases, glycosidases, etc.) could also be immobilized to address all mAb CQAs.

## Data Availability

The original contributions presented in the study are included in the article/Supplementary Material, and further inquiries can be directed to the corresponding author.
