# Supplementary material for: Monolithic Papain-Immobilized Enzyme Reactors for Automated Structural Characterization of Monoclonal Antibodies
Source: Front Mol Biosci. 2021 Nov 9;8:765683. doi: 10.3389/fmolb.2021.765683 (PMC8630785; doi:10.3389/fmolb.2021.765683)
Supplement: Supplementary file 1 [file DataSheet1.DOCX]

Supplementary Material

**Supplementary Figure 1.** Chromatograms of RTX samples digested by papain overnight in solution (red) and in the silica (blue) and polyHIPE IMERs (light blue); the time range including digested and intact RTX peaks **(A)** and a zoom in the digested RTX region **(B)** are shown. In **B**, each peak is labelled with the correspondent mAb fragment assignment by LC-MS analyses.

**Supplementary Figure 2.** Deconvoluted mass spectra obtained for each mAb fragment (Fc/2-K, pLC, Fc-K, pFab, pFab-LC, respectively) in the LC-MS analyses of RTX samples digested by papain overnight in solution **(A)** and in silica **(B)** and polyHIPE IMERs **(C)**.

**Supplementary Figure 3.** Chromatograms of RTX sample before (red) and after (blue) incubation in the silica **(A)** and polyHIPE **(B)** IMERs included in the on-line system.
